# Subjective experience of time in dementia with Lewy bodies during COVID-19 lockdown

**DOI:** 10.1007/s12144-021-01811-7

**Published:** 2021-05-08

**Authors:** Dylan Torboli, Giovanna Mioni, Cinzia Bussé, Annachiara Cagnin, Antonino Vallesi

**Affiliations:** 1 Department of General Psychology, University of Padova, Padova, Italy; 2 Departiment of Neuroscience, University of Padova, Padova, Italy; 3 Department of Neuroscience & Padova Neuroscience Center, University of Padova, Padova, Italy; 4 IRCCS San Camillo Hospital , Venice, Italy

**Keywords:** Dementia, Lewy bodies, Time perception, COVID-19, Lockdown

## Abstract

Dementia with Lewy bodies (DLB) is a neurodegenerative disease characterized by cognitive, behavioral and motor symptoms and has a more challenging clinical management and poorer prognosis compared to other forms of dementia. The experience of lockdown leads to negative psychological outcomes for fragile people such as elderly with dementia, particularly for DLB, causing a worsening of cognitive and neuropsychiatric symptoms. Since an individual’s feeling of time passage is strongly related to their cognitive and emotional state, it is conceivable to expect alterations of this construct in people with DLB during such a difficult period. We therefore assessed the subjective experience of the passage of time for present and past time intervals (Subjective Time Questionnaire, STQ) during the lockdown due to coronavirus disease (COVID−19) in 22 patients with DLB (17 of which were re-tested in a post-lockdown period) and compared their experience with that of 14 caregivers with similar age. Patients showed a significantly slower perception of present and past time spent under lockdown restrictions. We argue that these alterations might be related to the distinctive features of DLB and their exacerbation recorded by the patients’ caregivers during the period of lockdown, though our results show that the patients’ experience of time passage in a post-lockdown period remained similarly slow. Overall, we show an impairment of the subjective perception of time passage in DLB tested during the COVID-19 lockdown.

**Supplementary Information:**

The online version contains supplementary material available at 10.1007/s12144-021-01811-7.

## Introduction

The notion of time represents a fundamental component of human experience (Paton & Buonomano, 2018). The temporal structure of surrounding stimuli defines the way information is perceived, experienced, and remembered (Merchant et al., 2013) and time-dependent cognition enables us to interact with an ever-changing environment (Finnerty et al., 2015).

The concept of subjective time refers to the way individuals perceive and evaluate their personal time during their lifespan (Gabrian et al., 2017) and one of its primary dimensions is time awareness, which can be defined as the subjective impression of time as moving quickly or slowly (Wittman & Lehnhoff, 2005). Unlike physical time, the subjective experience of time passage can be affected by variations in external stimulation (Droit-Volet & Meck, 2007; Zakay & Block, 1997) and by the individual’s emotional and cognitive state (Droit-Volet, 2013; Droit-Volet & Gil, 2009; Jokic et al., 2018; Teixeira et al., 2013; Wittman, 2009). Passage of time judgments (POTJs; i.e., the construct addressed by this work) are judgments of how quickly or slowly time seems to pass relative to “normal” situations for an individual. They are not the same construct as duration judgments (DJs; i.e., judgments relative to the duration of a stimulus or an event) (Droit-Volet & Wearden, 2016; Wearden et al., 2014). While the latter typically address short time intervals ranging from seconds to minutes, POTJs cover larger time spans and do not require a comparison between a subjective estimate and clock time (Wittman & Lehnhoff, 2005). Though the cognitive mechanisms underlying the generation of POTJs are still unclear, we can assume that they rely on certain psychological processes, namely preserved vigilance, attention, retrospective memory, decision-making, awareness of the self, intact notion of the concept of time. Evidence from Wearden et al. (2014) also suggests that POTJs are related to hedonic variables, with positive states (enjoyment, excitement, liking, engagement) being associated with faster POTJs and negative ones (boredom, sadness, annoyance, fatigue) with slower POTJs (Wearden, 2015).

There is no human clinical condition that can be defined solely as a disorder of timing and time perception per se, although distortions and perturbations in timing ability are present in many populations and may be associated with different developmental, cognitive and behavioral profiles (Allman & Meck, 2011). As a matter of fact, individuals with psychiatric or neurologic conditions often exhibit difficulty in perceiving and organizing time, frequently due to disorders on attention, memory and related to neurotransmitters dysregulation affecting dopamine and acetylcholine systems (Marinho et al., 2017).

Amongst the many existing neurodegenerative disorders, dementia with Lewy bodies (DLB) stands out for its challenging management and poorer prognosis (Mueller et al., 2017) due to the mixture of neuropsychiatric, motor and autonomic symptoms. Compared with Alzheimer’s Disease (AD), a diagnosis of DLB is associated with steeper cognitive decline (Kramberger et al., 2017), earlier admission in nursing homes (Rongve et al., 2013), poorer quality of life (Bostrom et al., 2007), greater caregiver burden (Svendsboe et al., 2016) and increased mortality (Price et al., 2017; Williams et al., 2006).

So far, timing abilities have not yet been extensively investigated in DLB patients. Only two studies have been conducted. The first is a pilot study by Lesimple et al. (2016) which showed impairment in the perception of rhythmic variations and verbal time estimation in 7 patients with DLB as compared with healthy controls. The other study (Matar et al., 2019) tested 25 patients with probable DLB and 14 older controls in a simple time perception paradigm, finding altered temporal processing of target intervals that correlated with cognitive fluctuations in the DLB group. To the best of our knowledge, the dimensions of time awareness and POTJs have never been examined in people with DLB. Beyond this gap in the current literature, our choice of studying the experience of time in a group of people with DLB was motivated by anatomical and clinical evidence concerning this disease. Recent literature (Fathy et al., 2018; Philippi et al., 2020; Roquet et al., 2017) agrees on an involvement of insular cortex atrophy in the early stages of DLB. Though with some controversy, this structure has been attributed a role in the awareness of subjective time (Craig, 2009; Wittmann et al., 2010; Wittmann et al., 2010). Moreover, DLB neurodegeneration is known to involve subcortical structures such as the putamen to a greater extent compared to other forms of dementia such as AD (Cousins et al., 2003; Mak et al., 2014): nigrostriatal dopaminergic neurons are known to subserve various cognitive functions implicated in time perception, namely memory, attention, decision-making and perceptual capacity (Marinho et al., 2017), and striatal areas are unanimously considered critical for the processing of time (Coull & Nobre, 2008; Matell & Meck, 2004; Merchant et al., 2013; Merchant & de Lafuente, 2014; Paton & Buonomano, 2018). Therefore, from an anatomo-functional perspective, it is plausible to expect distortions of time perception in DLB even in the early stages of the disease. Worth noting, during the implementation of the present study, Italy was undergoing a public health crisis due to the global epidemic from coronavirus disease (COVID-19) caused by the severe acute respiratory syndrome coronavirus 2 (SARS-COV − 2), that started in December 2019 in the city of Wuhan, China. Starting from March 10th 2020, the Italian Government issued a series of decrees that imposed lockdown to all the citizens in the country until May 3rd. Lockdown is a period of restriction of movement of people, who are confined to their homes and socially isolated. Lockdown is a severe measure imposed by governments as an attempt to reduce or block the chain of transmission of a contagious disease and contain the spread of infections. Although necessary, this measure and similar ones such as quarantine are often lived as an unpleasant experience that brings along negative outcomes in terms of physical and mental health for those who undergo it (Brooks et al., 2020). Fear, anxiety, loss of control and a feeling of being trapped are among the most commonly reported effects (Rubin & Wessely, 2020), but also symptoms of post-traumatic stress disorder and suicides have been observed (Brooks et al., 2020).

In the case of people with mental health conditions, they could be markedly more susceptible to the emotional responses triggered by the pandemic and by the experience of lockdown, with consequent worsening of already existing neuropsychiatric symptoms or onset of new behavioral disorders because of a high susceptibility to stress compared with general population (Yao et al., 2020), particularly for people with dementia, who might feel abandoned and become withdrawn (Wang et al., 2020), due to the abrupt lack of personal support and social interactions.

Among the many variants of dementia, it has been shown that patients with DLB, given their significantly disabling clinical profile, might be particularly vulnerable to the psychological outcomes of the pandemic, leading to a worsening of their symptoms and of their caregivers’ burden (Cagnin et al., 2020; Migliaccio & Bouzigues, 2020). In general, apathy, agitation and anxiety are the most frequently reported worsening symptoms during quarantine in patients with dementia (Simonetti et al., 2020), particularly in the case of DLB patients who also showed an increased risk of worsening hallucinations and sleep disorders (Cagnin et al., 2020). Lockdown and quarantine may be indeed considered as ecological models of the effects of deprivation of multidimensional stimulation, leading to a global down-regulation of cognitive, physical and emotional domains, with increased apathy as one of its main manifestations (Cagnin et al., 2020; Simonetti et al., 2020).

In this context, since the sense of time is determined by an intricate relationship between cognitive functions and temporary emotional states (Wittmann, 2009), it is likely that the subjective perception of time will be particularly impaired in patients with DLB during the COVID−19 pandemic, due to the combined effect of their clinical condition and of the experience of lockdown. The subjective experience of time passage during the restrictive measures adopted to contrast the COVID-19 pandemic has already been addressed by other researchers (Cellini et al., 2020; Droit-Volet et al., 2020; Martinelli et al., 2021; Ogden, 2020) who found a slowing down of time experience predicted mainly by feelings of boredom and lack of activity, sleeping difficulties, decreased happiness and lower arousal levels. While these studies involved large samples of healthy individuals, the experience of time of people with dementia during lockdown remains under-explored.

Starting from these assumptions, the main goal of this study is to describe the subjective experience of time passage in a group of patients with a diagnosis of probable DLB examined during the period of lockdown from COVID-19, with the expectation to find an altered perception of time in comparison with healthy controls of similar age. In order to do so, we employed the Subjective Time Questionnaire (STQ; Wittman & Lehnhoff, 2005), a tool that assesses how the passage of time is typically experienced in everyday life and how quickly past time intervals are judged to have passed (Mioni et al., 2020), but also the tendency to perceive time at disposal either as scarce and restricted or abundant and dilated. The scores obtained by the patients were compared with the ones obtained by a group of caregivers of the same patients (thus with a similar age and sharing the same environment) in order to isolate and verify a potential differential effect on the subjective perception of time passage of these two separate conditions. We also re-interviewed the majority of the same group of DLB patients in a post-lockdown period: this was done to verify if a reduction in the restrictions imposed by the lockdown would be accompanied by a decreased impairment in the subjective perception of time.

Based on previous findings (Cellini et al., 2020; Droit-Volet et al., 2020; Martinelli et al., 2021; Ogden, 2020; Wearden, 2015), the leading hypothesis was that the negative states induced by the experience of lockdown would cause a subjective slowing down of the perception of time passage in both groups, and that this would be especially true for the DLB group due to an exacerbation of their neuropsychiatric symptoms caused by a maladaptation to environmental changes and a higher susceptibility to stress.

## Method

### Participants

Thirty-six individuals participated in the present study: 22 diagnosed with probable DLB according to updated McKeith criteria (McKeith et al., 2017), selected among the patients who refer to the outpatient clinics for memory disorders of the neurology department of Padua’s Hospital; 14 healthy caregivers chosen from the family members who are mostly engaged in the care of the recruited patients. Three patients and 4 caregivers that were initially screened did not join the research for reasons of indisposition, refusal, or severity of deterioration incompatible with the administration of the questionnaire. All patients lived in their own houses, most of them (75%) with their caregiver. Each patient had a recent cognitive screening (MMSE <6 months) certifying the existence of an ongoing cognitive impairment (mean MMSE raw score = 25.13 ± 3.04 SD). Furthermore, the STQ was re-administered to 17 out of the initially interviewed 22 DLB patients (5 of which were indisposed to participate again) in a post-lockdown period.

The demographic characteristics of the recruited samples are presented in Table 1.

**Table 1:** Demographic characteristics of the samples

	DLB *n* = 22 (*M* ± *SD*)	Caregivers *n* = 14 (*M* ± *SD*)	DLB-POST *n* = 17 (*M* ± *SD*)
Age	75.40 ± 5.52	71.64 ± 10.89	75.76 ± 5.61
Education	11.04 ± 4.06	10.14 ± 3.84	10.52 ± 3.93
Gender	m = 17 (77%)	m = 2 (14%)	m = 13 (76%)
MMSE score	25.13 ± 3.04	N.A.	25.70 ± 3.01

### Procedure

After obtaining a preliminary verbal informed consent, an experimenter interviewed each participant by telephone call. The first run of telephone interviews was carried out in the timespan between 04/15/2020 and 04/30/2020, a period in which all Italian citizens were living under the restrictive lockdown measures, while the second run (involving the DLB group only) was accomplished between 07/06/2020 and 07/10/2020, two months after the end of the lockdown. Each volunteer underwent the telephone administration of the STQ, which was filled out in real time by an examiner and requested an average administration time of ten minutes per participant. The study was approved by the Ethics Committee of Padua’s Hospital.

### Materials

In this study, we used a shortened Italian version of the “Subjective Time Questionnaire” (STQ), a tool developed by Wittman and Lehnhoff (2005) for the assessment of the individual experience of time through the analysis of personal judgments on the passage of time.

The questionnaire is composed by two groups of items to be answered on Likert scale:

1. “Personal Time Experience of Present and Past”. This part includes items related to the daily experience of the passage of time and retrospective evaluations of past time intervals, to which respondents are required to answer on a five-anchors Likert scale ranging from −2 = “very slowly” to +2 = “very fast” (Mioni et al., 2020). The two questions covering the perception of present time (“How fast does time usually pass for you?” and “How fast do you expect the next hour to pass?”) differ in that the former reflects a view of the passage of time in general, while the latter represents a more transient and state-like momentary perception of time. These two questions are collapsed to form the STQ-present index. The second set of items, which require retrospective judgments of long intervals and past life periods, includes four questions that investigate how fast last week, month, year, and 10 years passed for the individual. In the original version of the questionnaire, the mean value of these four judgements forms the STQ-past index (Mioni et al., 2020). For this research, though, we agreed on setting a distinction between two indices in order to differentiate the subjective perception of recent past time lived under lockdown restrictions (STQ-lockdown, formed by items 3 “How fast did the previous week pass for you?” and 4 “How fast did the previous month pass for you”?) from the perception of more remote past intervals lived in “normal” life conditions (STQ-past, formed by items 5 “How fast did the previous year pass for you?” and 6 “How fast did the previous 10 years pass for you?”). The original version of the questionnaire also includes four questions related to the subjective perception of time of the periods of childhood, adolescence, young adulthood and adulthood that have been excluded from this research, since it is a kind of representation that would be difficult to achieve by patients with DLB.
2. “Statements on Subjective Time Experience”. This part consists of statements on the subjective experience of the time about which respondents must express their degree of agreement on a five-anchors Likert scale ranging from 1 = “strong rejection” to 5 = “strong approval”. These ten items are balanced between statements that refer either to a feeling of compression/scarcity of time (e.g., “I often feel time is running out”), forming the Time Pressure index, or to a feeling of expansion/abundance of time (e.g., “I often think that time just does not want to pass”), forming the complementary Time Expansion index. The original version of the questionnaire also includes six metaphors that describe time passage either as fast (e.g., “Time is a speeding train) or slow (e.g., “Time is a quiet, motionless sea”). These items were excluded from the present study as they are of little relevance to our purposes, but also because they may not be fully understood by the group of DLB patients.

The version of the STQ (both in English and Italian) used for this work is shown in the Appendix.

### Statistics

Statistical analyses were performed using the open-source software JASP (version 0.12.2; https://jasp-stats.org). Demographic characteristics and STQ scores were confronted between groups using the Mann-Whitney non-parametric U test for independent samples. A Wilcoxon signed-rank test was performed to evaluate differences in the STQ indices within the 17 DLB patients that were re-tested in a post-lockdown period. We also calculated the Spearman correlation index (*r*) to test the existence of a possible relationship between the MMSE scores and the values of the STQ indices in the DLB group. Statistical significance was set for *p* values <0.05.

## Results

### Demographic Features

Performed analyses (see Table 2) show that patients with DLB, when compared with their caregivers, stand out for their significantly greater number of male members. Conversely, DLB and DLB-POST groups share very similar demographic characteristics, being mostly composed by the same members (apart from those patients lost due to attrition).

**Table 2:** Comparison of demographic characteristics with Mann-Whitney U test. Bold values show significant results (*p* < .05)

	DLB vs Caregivers	DLB vs DLB-POST
Age	0.053	0.809
Education	0.952	0.794
Gender	**< 0.001**	0.969

### Subjective Experience of Time

**Fig. 1:**
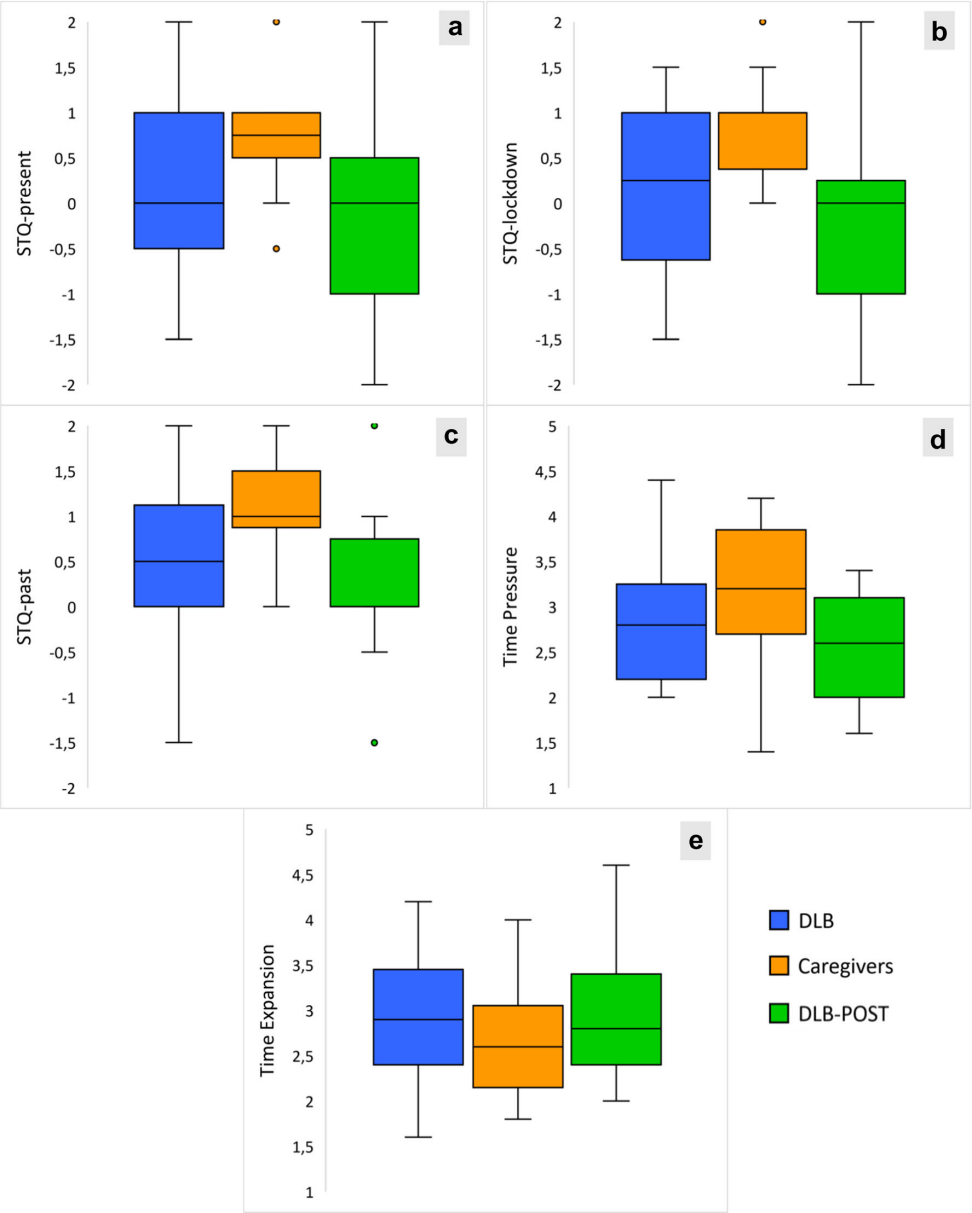
**a** Distribution of the samples on STQ-present index. **b** Distribution of the samples on STQ-lockdown index. **b** Distribution of the samples on STQ-past index. **d** Distribution of the samples on Time Pressure index. **e** Distribution of the samples on Time Expansion index. DLB = Dementia with Lewy Bodies. The small dots represent outliers in the distribution

The median (*Mdn*) and inter-quartile range (*IQR*) of the scores achieved by the recruited samples on the indices of the STQ are reported in the Supplementary Material (Table S1). Table S2 shows the *p* values obtained from the comparison between groups with a Mann-Whitney U test. Figure 1 displays the distribution of the scores obtained by our samples on the STQ indices.

Overall, results show that DLB patients tended to provide neutral answers (with median scores equal or very close to 0; see Supplementary Table S1), possibly indicative of a subjective experience of time that is neither fast nor slow. Statistically significant differences between the two groups were found on the STQ-present index (*Mdn* DLB = 0.00; *Mdn* caregivers = 0.75; *U* = 215.5; *p* = 0.044) and STQ-lockdown index (*Mdn* DLB = 0.25; *Mdn* caregivers = 1.00; *U* = 220.5; *p* = 0.028), with an observed subjective slowing of time perception relative to present and recent past (i.e., last week and month spent during the lockdown) in the DLB patients compared with their caregivers. On the other hand, the two groups differ only mildly in the perception of remote past periods (STQ-past; *Mdn* DLB = 0.50; *Mdn* caregivers = 1.00; *U* = 206; *p* = 0.088). No differences emerged between any of the groups on Time Pressure and Time Expansion indices. Therefore, during COVID-19 lockdown, patients with DLB generally tend to report a slower subjective experience of time than their caregivers, with no different feelings of time pressure or expansion.

Finally, no statistically significant differences emerged from the comparison between the DLB group under lockdown and part of the same group examined in a post-lockdown period, a result confirmed by a within-subjects comparison performed with a Wilcoxon signed-rank test (see Supplementary Table S3). Therefore, it seems that people with this type of dementia show a similarly impaired subjective perception of time passage regardless of whether they are under lockdown restrictions or not. In conclusion, the feeling that time passes slower did not decrease in a post-lockdown period for the DLB group, suggesting that they experience time as passing more slowly in general.

### Cognitive Impairment

Spearman’s correlation coefficient did not show any significant relationship (all *p* > .22) between the *Mini Mental State Examination* (MMSE) score and the value of the STQ indices obtained by the group of DLB patients (Supplementary Table S4). Therefore, there seems to be no relationship between the severity of cognitive impairment and the subjective perception of time in this sample.

## Discussion

This study shows that patients with DLB are characterized by a subjective perception of time that is significantly slower compared to that of their caregivers during the period of lockdown from COVID-19. More in detail, this slowing is mainly observed in the case of subjective judgments on the passage of present and recent past time (STQ-present and STQ-lockdown, see Supplementary Material), while it declines when longer time spans are addressed (STQ-past). In general, these effects may reflect the impact of the disease on the investigated construct (i.e., the subjective perception of the passage of time), the extent of which might grow for the time periods lived under lockdown restrictions. A closer look at the DLB data shows a tendency to provide “timeless” POTJs. As suggested by an anonymous reviewer, this time-related “apathy” might have different, not necessarily mutually exclusive explanations that need to be investigated more directly by future research. DLB patients might have impaired decision-making and would happily choose the neutral option, or they might be incapable of forming metacognitive representations of long time spans because of impaired memory processes. Moreover, they might be unaware of time or lost knowledge of notions related to time. Symptoms of altered awareness of time have already been found among other types of dementias (Requena-Komuro et al., 2020), and it is not surprising that such an impairment is present in DLB patients as well.

The fact that patients with DLB actually feel time as passing neither fast nor slow (both during lockdown and soon after) is actually also consistent with a common feature of DLB, namely apathy, which has been found to be exacerbated during lockdown by a large multi-center study (Cagnin et al., 2020) that also involved 360 caregivers of people with DLB, including most of the very same patients of the present study. This research found that DLB patients had the highest frequency of behavioral and psychological symptoms during the lockdown among all the types of dementias that were studied. In particular, DLB stood out for the worsening of sleep disorders, hallucinations, apathy, anxiety and depression (Cagnin et al., 2020). These data may account for the observed relative slowing down of time perception in the DLB group with respect to their caregivers during lockdown. Previous studies (Wearden, 2015) highlighted the influence of hedonic variables on POTJs, and most recent research (Martinelli et al., 2021) also found that sleep disturbances and decreased happiness contribute to explain the feeling of a slowing down of time during lockdown.

No differences were found between our groups on the values of Time Pressure and Time Expansion indices, suggesting that neither DLB nor the experience of lockdown exert an influence over the perceived amount of time at one’s disposal or the feeling of a restricted/dilated flow of time.

The fact that the responses to the STQ provided by the DLB patients have not undergone significant changes following the end of the lockdown deserves further discussion. It may suggest that DLB patients feel present and recent past time as passing relatively slower in general and that such experience does not affect, at least in people with this diagnosis, the subjective perception of time, but also that the alterations found exclusively depend on the disease. On the other hand, the post-lockdown period during which patients were retested cannot be considered a normal living condition, considering that the pandemic was still ongoing (with elderly individuals adopting measures of self-lockdown) and that the adverse psychological effects of the lockdown could still persist. Hence, from the results of this study it is not possible to disentangle the distinct contributions of lockdown and of the disease on determining the subjective perception of time in the examined group.

Ultimately, the absence of significant correlations between the MMSE scores and the STQ indices suggests that the peculiar subjective experience of time of the DLB patients does not depend on the severity of their general cognitive impairment, but rather on the distinctive features of the disease (likely apathy, confusion, impaired attention and memory, fluctuating arousal and cognition).

## Limitations

The limitations of this research are mainly identifiable in the composition of the samples. Among these, the most evident is the low number of participants, although this is reasonable considering the circumstances of implementation of this study and the specificity of the selected diagnosis. Secondly, gender distribution is also not equally balanced between the DLB group, where male sex prevails, and their caregivers, who are mainly represented by females. Ultimately, caregivers do not have a MMSE that certifies their cognitive integrity, so we can only indirectly assume that these individuals are cognitively healthy.

In light of the above limitations, it is clear that this work would benefit from a greater number of participants, as well as more evenly distributed samples in terms of demographic characteristics, in order to allow more reliable comparisons.

## Conclusion

In the present research we studied the subjective experience of time during COVID-19 lockdown in a group of people with DLB, using a shortened version of the *Subjective Time Questionnaire* developed by Wittman and Lehnhoff (2005), and compared it with the perception of a group of similar age caregivers. Patients with a DLB diagnosis tend to report that time does not pass either slowly or quickly for them, and this perception of time passage is slower than the one reported by their caregivers in their responses to the questionnaire. Therefore, it seems that patients with DLB, compared with healthy controls, perceive time as passing relatively slower during the lockdown due to COVID-19 pandemic. We argue that this subjective experience may be the consequence of a cognitive-emotional slowdown resulting from the joint effect of the clinical features of the disease and the adverse influence of the experience of lockdown on the behavioral and psychological profile of the patients. Indeed, the literature (Brooks et al., 2020) reports how social isolation from quarantine or lockdown is frequently associated with negative psychological outcomes including stress, fear, anxiety, mood disorders, apathy and insomnia which, in fragile people such as patients with dementia, can easily lead to an exacerbation of the symptomatology (Cagnin et al., 2020; Simonetti et al., 2020), likely affecting their awareness of time as well. In the specific case of the patients recruited in this study, it would seem that they tend to live the passage of time during the lockdown with detachment, finding refuge in a personal temporal dimension. Consistently with these findings and interpretations, the DLB patients do not report feelings of time pressure or time dilation.

Though aware of the limitations of this research, we provide a photograph, albeit incomplete and imperfect, of how DLB and the caregiver status of patients with DLB can interact with the isolation imposed by the lockdown restrictions on the subjective perception of time. To our best knowledge, it is the first time that this construct has been studied in this type of patients. The obtained results represent an initial step in the identification of the factors that contribute to the individual experience of time in DLB.

### Supplementary Information

ESM 1 (DOCX 18 kb)

## Data Availability

The anonymized datasets generated and analysed during the current study are available from the corresponding author on reasonable request.

## Appendix Subjective time questionnaire items and scoring (Wittman & Lehnhoff, 2005)

**Table:**

English version Subjective time questionnaire (Wittman & Lehnhoff, 2005)					
*Personal time experience of present and past* *(−2 = very slowly, −1 = slowly, 0 = neither fast nor slow, 1 = fast, 2 = very fast)*					
	-2	-1	0	1	2
*i1)* How fast does time usually pass for you?					
*i2)* How fast do you expect the next hour to pass?					
*i3)* How fast did the previous week pass for you?					
*i4)* How fast did the previous month pass for you?					
*i5)* How fast did the previous year pass for you?					
*i6)* How fast did the previous 10 years pass for you?					
*Statements on subjective time experience* *(1 = strong rejection, 2 = rejection, 3 = neutral, 4 = approval, 5 = strong approval)*					
*ii1)* I haven’t enough time to complete my tasks	1	2	3	4	5
*ii2)* My time seems empty					
*ii3)* I often think that time just does not want to pass					
*ii4)* I often feel time pressure					
*ii5)* I often haven’t enough time to devote myself to important things					
*ii6)* I often feel bored					
*ii7)* I often think time is running out					
*ii8)* I have a lot of time					
*ii9)* I often have spent my time without doing anything					
*ii10)* I have to establish my priorities, because I cannot do all the things I would like to do					
Italian version Questionario della percezione soggettiva del tempo (Mioni et al., 2020)					
*Esperienza temporale personale del presente e del passato* *(−2 = molto lentamente, −1 = lentamente, 0 = né veloce né lento, 1 = velocemente, 2 = molto velocemente)*					
	-2	-1	0	1	2
*i1)* Solitamente quanto veloce passa il tempo per te?					
*i2)* Quanto velocemente ti aspetti che passerà la prossima ora?					
*i3)* Quanto velocemente è trascorsa la precedente settimana per te?					
*i4)* Quanto velocemente è trascorso lo scorso mese per te?					
*i5)* Quanto velocemente è passato lo scorso anno per te?					
*i6)* Quanto velocemente sono trascorsi per te gli ultimi 10 anni?					
*Enunciati sull’esperienza soggettiva del tempo* *(1 = fortemente in disaccordo, 2 = in disaccordo, 3 = neutrale, 4 = d’ accordo, 5 = fortemente d’accordo)*					
*ii1)* Non ho abbastanza tempo per completare ciò che ho da fare	1	2	3	4	5
*ii2)* Il mio tempo non è completamente pieno di cose da fare					
*ii3)* Spesso penso che il tempo non voglia passare					
*ii4)* Sento spesso la pressione del tempo					
*ii5)* Spesso non ho abbastanza tempo per dedicarmi alle cose che giudico importanti					
*ii6)* Spesso mi sento annoiato					
*ii7)* Spesso penso che il tempo stia “correndo via”					
*ii8)* Ho un sacco di tempo a disposizione					
*ii9)* Spesso devo usare il mio tempo facendo qualcosa					
*ii10)* Devo stabilire delle priorità, poiché non posso fare tutte le cose che mi piacerebbe fare					
