# Supplementary material for: Subjective experience of time in dementia with Lewy bodies during COVID-19 lockdown
Source: Curr Psychol. 2021 May 8;42(6):4653–62. doi: 10.1007/s12144-021-01811-7 (PMC8105146; doi:10.1007/s12144-021-01811-7)
Supplement: Supplementary file 1 — (DOCX 18 kb) [file 12144_2021_1811_MOESM1_ESM.docx]

**Supplementary Material: Torboli et al.**

|  | DLB  ( *Mdn*; *IQR* ) | Caregivers  ( *Mdn*; *IQR* ) | DLB-POST  ( *Mdn*; *IQR* ) |
| --- | --- | --- | --- |
| STQ-PRESENT | 0.00; 1.375 | 0.75; 0.50 | 0.00; 1.50 |
| STQ-Lockdown | 0.25; 1.25 | 1.00; 0.50 | 0.00; 1.00 |
| STQ-PAST | 0.50; 1.00 | 1.00; 0.50 | 0.00; 0.50 |
| Time Pressure | 2.80; 0.95 | 3.20; 0.95 | 2.60; 1.00 |
| Time Expansion | 2.90; 1.00 | 2.60; 0.80 | 2.80; 0.80 |

*Table S1: Median (Mdn) and inter-quartile range (IQR) values on STQ indices.*

|  | DLB vs Caregivers  ( *p* ) | DLB vs DLB-POST  ( *p* ) |
| --- | --- | --- |
| STQ-PRESENT | **0.044**** | 0.741 |
| STQ-Lockdown | **0.028***** | 0.261 |
| STQ-PAST | 0.088* | 0.204 |
| Time Pressure | 0.138 | 0.340 |
| Time Expansion | 0.272 | 0.798 |

*Table S2: between groups comparisons on the STQ indices with Mann-Whitney U test;*

** = p < 0.1; ** = p < 0.05; *** = p < 0.03.* *Bold values show significant result.*

|  | DLB pre vs post  *( p )* |  |
| --- | --- | --- |
| STQ-PRESENT | 0.927 |  |
| STQ-Lockdown | 0.384 |  |
| STQ-PAST | 0.340 |  |
| Time Pressure | 0.244 |  |
| Time Expansion | 0.172 |  |

*Table S3: comparison on STQ indices within DLB participants who were tested before and after lockdown using Wilcoxon signed-rank test; * = p < 0.1; ** = p < 0.05; *** = p < 0.03.* *Bold values show significant result.*

|  | MMSE | |  |
| --- | --- | --- | --- |
|  | **Spearman’s rho** | ***p*** |  |
| STQ-PRESENT | - 0.132 | 0.559 |  |
| STQ-Lockdown | - 0.123 | 0.584 |  |
| STQ-PAST | 0.145 | 0.521 |  |
| Time Pressure | 0.273 | 0.220 |  |
| Time Expansion | - 0.135 | 0.549 |  |

*Table S4: correlations between MMSE score and STQ score in the DLB group using Spearman coefficient;*

** = p < 0.1; ** = p < 0.05; *** = p < 0.03.* *Bold values show significant result.*
